# Vitamin D Status and Selected Metabolic Parameters in Salt Mine Workers: A Cross-Sectional Study

**DOI:** 10.3390/nu18081287

**Published:** 2026-04-19

**Authors:** Malwina Pietrzak, Katarzyna Sobczak, Katarzyna Domaszewska

**Affiliations:** 1 Department of Physiology, Faculty of Health Sciences, University of Physical Education, Królowej Jadwigi Street 27/39, 61-871 Poznań, Poland; m.pietrzak@awf.poznan.pl; 2 Department of Swimming and Water Lifesaving, Faculty of Sports Science, Poznan University of Physical Education, Królowej Jadwigi Street 27/39, 61-871 Poznań, Poland; ksobczak@awf.poznan.pl

**Keywords:** vitamin D, 25-hydroxyvitamin D, miners, occupational health, insulin resistance, HOMA-IR, lipid metabolism, glucose metabolism

## Abstract

**Background:** Vitamin D deficiency has been implicated in disturbances of glucose and lipid metabolism, particularly in occupational groups with limited sunlight exposure. This study aimed to examine the association between serum 25-hydroxyvitamin D [25(OH)D] concentrations and markers of carbohydrate and lipid metabolism in salt mine workers. **Methods:** This cross-sectional study involved 62 male salt miners (aged 25–63 years), stratified by work depth (surface, ≤750 m, and >750 m). Anthropometric characteristics, body composition, cardiorespiratory fitness (VO_2max_), and biochemical parameters were assessed. Blood analyses included fasting glucose, insulin, lipid profile, TSH, and 25(OH)D. Insulin resistance was evaluated via the HOMA-IR index. **Results:** The cohort exhibited a high prevalence of overweight and obesity (mean BMI > 28 kg/m^2^). Significant differences in VO_2max_ were observed between groups (*p* < 0.05). Elevated fasting glucose (>100 mg/dL) was observed in 47% of participants, and 22% presented HOMA-IR values > 2.5. In the regression model, vitamin D supplementation was the strongest predictor of 25(OH)D levels, explaining 25.5% of its variance. The addition of HDL cholesterol increased the explained variance to 35.6%, whereas HOMA-IR contributed an additional 3.9% (*p* = 0.094). **Conclusions:** In salt miners, insufficient vitamin D status coexists with excess adiposity and impaired glucose homeostasis. Serum 25(OH)D was more strongly associated with supplementation and HDL-C than with HOMA-IR. These findings suggest that monitoring vitamin D status is relevant in the occupational health evaluation of this group.

## 1. Introduction

In recent years, vitamin D has attracted growing scientific interest due to its important role not only in bone metabolism, but also in carbohydrate and lipid homeostasis [1]. Vitamin D is a steroid hormone synthesized in the skin following exposure to ultraviolet radiation and may also be obtained from dietary sources or supplementation [1]. Vitamin D deficiency has been associated with numerous adverse health outcomes, including cardiovascular diseases, infections, cancer, diabetes, and autoimmune disorders [2]. Increasing evidence also suggests that vitamin D is involved in the regulation of glucose and lipid metabolism and may therefore contribute to the development of metabolic disturbances [3].

Vitamin D deficiency has also been linked to an increased risk of metabolic syndrome (MetS), a cluster of abnormalities including insulin resistance, dyslipidemia, hypertension, obesity, and disturbances in glucose metabolism, all of which contribute to the development of type 2 diabetes and atherosclerotic cardiovascular disease [4]. Metabolic syndrome is characterized by increased visceral adiposity, elevated blood pressure, dysglycemia, hypertriglyceridemia, and reduced high-density lipoprotein (HDL) cholesterol concentrations [4]. One of its key pathophysiological components is insulin resistance, which is associated with obesity, impaired glycemic regulation, and increased cardiovascular risk [5,6]. In clinical and epidemiological studies, insulin resistance is commonly assessed using the HOMA-IR (homeostatic model assessment of insulin resistance) index, calculated on the basis of fasting glucose and insulin concentrations. Elevated HOMA-IR values indicate an excessive insulin response relative to glucose levels and are widely used as markers of impaired insulin sensitivity [7,8].

Because vitamin D is a fat-soluble compound, its relationship with adipose tissue is of particular importance. The presence of vitamin D receptors (VDR) in adipose tissue and their responsiveness to calcitriol concentrations support the role of vitamin D in the regulation of adipose tissue metabolism [9]. Obesity is associated with increased adipose tissue mass and impaired adipocyte function, and calcitriol has been shown to modulate adipocyte metabolism, including lipogenesis [9]. Accordingly, systemic vitamin D deficiency is more frequently observed in individuals with obesity than in those with normal body weight [10]. Low calcidiol concentrations may also lead to increased serum parathyroid hormone (PTH) levels and secondary hyperparathyroidism. Despite its important biological role, vitamin D deficiency affects nearly one billion people worldwide, regardless of age or ethnicity. Serum 25-hydroxyvitamin D [25(OH)D], the major circulating form of vitamin D produced through hepatic hydroxylation, is considered the most reliable indicator of vitamin D status.

These issues may be particularly relevant in occupational groups exposed to limited sunlight and circadian disruption, such as miners. Shift work, especially night work, is an inherent component of mining and disrupts the sleep–wake cycle and circadian rhythm [11]. Circadian rhythm disturbances affect the secretion of several hormones, including prolactin, melatonin, serotonin, glucocorticosteroids, adrenocorticotropic hormone, and corticotropin-releasing factor, and may consequently increase the risk of metabolic syndrome [12,13,14]. Efficient energy metabolism is essential for normal physiological functioning, whereas sleep deprivation significantly alters endocrine and metabolic parameters associated with diabetes, obesity, and other metabolic disorders. Reduced melatonin secretion resulting from exposure to light at night may impair lipid metabolism and increase sympathetic nervous system activity [15]. Shift workers have been shown to present higher triglyceride concentrations and lower HDL cholesterol levels, both of which are directly associated with metabolic disturbances [16].

Melatonin also plays a significant role in antioxidant defense. At the same time, long working hours may shorten the time available for rest and recovery and may themselves constitute a chronic psychological stressor [17], which in the long term may contribute to the development of cardiovascular diseases [18]. Moreover, shift workers have been reported to exhibit elevated levels of inflammatory markers, including C-reactive protein (CRP), as well as increased leukocyte counts, both of which are recognized risk factors for cardiovascular disease and ischemic stroke [19]. Shift work is also associated with poorer glycemic control, reflected by higher glycated hemoglobin (HbA1c) levels [20], and non-standard working hours may additionally promote unhealthy behaviors such as low physical activity and poor diet [21]. In miners, particularly those working underground, these risks may be further exacerbated by limited exposure to natural sunlight, which may substantially impair cutaneous vitamin D synthesis.

Although vitamin D has been implicated in metabolic regulation among shift workers, evidence in miners remains scarce. To date, only one study conducted in Serbian underground coal miners has addressed this issue [22]. Thus, a significant research gap remains regarding the relationship between vitamin D status and markers of lipid and carbohydrate metabolism in this specific occupational group. We hypothesized that lower 25(OH)D levels would be associated with insulin resistance and metabolic alterations in miners. Therefore, the aim of this study was to investigate the association between serum 25(OH)D concentration and markers of glucose and lipid metabolism in salt mine workers.

## 2. Materials and Methods

### 2.1. Participants

The study population consisted of 62 male salt miners aged 25–63 years. Data collection was conducted at the Rehabilitation and Treatment Center in Kłodawa between 24 February and 6 March 2020. Due to the nature of the underground workforce, the cohort was exclusively male. However, it should be noted that the number of women employed in the mining industry, particularly in underground work, is low, and no women were present in the study population. The study was conducted according to the Declaration of Helsinki and the National Statement of Intent as well as the Human Research Ethics Guidelines and was then approved by the IRB (Institute for Research in Biomedicine) at the Poznan University of Medical Sciences (19 June 2019; Ethics Approval Number: 695/19). Each recruited subject gave their written permission to take part after familiarizing themselves with the study protocol. The blood sampling and other tests carried out in the project did not affect the working regime of the mine.

The study population was divided into 3 groups. Group 1 comprised surface workers; group 2, underground workers (mining level > 750 m below the ground); group 3, underground workers (mining level ≤ 750 m below the ground), according to the structure of the salt mine. The current mining level is 750–780 m below the surface, and the level up to 750 m below the ground is no longer exploited. 

### 2.2. Anthropometric Measurements

The first anthropometric tests were performed each day. Participants were to refrain from drinking (except water) and eating for 3 h prior to the study.

Body height was measured using a Tanita HR-001 stadiometer (Tanita Corp., Tokyo, Japan). The measurement range was 0–2.07 m (0–81–1/2 inches), and the graduation was 1 mm (1/8 inch).

Body composition was measured using Bioelectric impedance analysis (BIA), which is commonly used in field surveys and also as a supplement to conventional anthropometry [23]. Body composition of men was estimated with bioelectric impedance analysis using TANITA BC-1000 BK + Program GMON Consumer (Tanita Corp., Tokyo, Japan), following the directions and procedures of the manufacturer. The unit provides a profile for the individual, including weight, BMI, fat mass (FM), muscle mass (MM), and visceral fat tissue (VFA).

### 2.3. Exercise Test

The exercise tests were conducted between 09:00 h and 11:00 h in an air-conditioned laboratory 2 h after consuming a light breakfast (one sandwich with butter and cheese; approximately 200 kcal). The exercise stress test laboratory was adequately equipped to provide advanced life support in the event of a cardiac arrest. The person executing the exercise test has authorization to perform the tests. Aerobic capacity was assessed with the modified Astrand–Rhyming protocol for predicting VO_2max_ by an ergometer Kettler DX1 Pro (Ense—Parsit, Germany), and heart rate (HR) was monitored using a Polar A-5 pulse meter (Polar Electro Oy, Kempele, Finland). The predicted VO_2max_ was read from the nomogram and multiplied by both the Astrand and the von Dobeln age correction factors. Oxygen consumption value is expressed in mL/kg/min [24].

### 2.4. Blood Analysis

Participants reported to the certified diagnostic laboratory ALAB (No AM 005) between 07.00 and 9.00 h after an overnight fast. Subjects were instructed to abstain from physical exercise between the final training bout and the final test. Blood samples (10 mL) were taken from the ulnar vein after a resting period of at least 10 min, using a S-Monovette syringe tube (Sarstedt AG & Co., Nümbrecht, Germany), then placed in tubes containing a clot activator, and centrifuged (1500 *g*, 4 °C, 4 min) (Universal 320R; Hettich Lab Technology, Tuttlingen, Germany).

The concentrations of glucose and lipid metabolism indices (TC, total cholesterol; TG, triglycerides; HDL-C, high-density lipoprotein cholesterol; LDL-C, low-density lipoprotein cholesterol) were determined with the enzymatic method. Insulin and TSH activity were measured by the non-isotopic carbonyl metalloimmunoassay (CMIA). The serum concentration of 25(OH)D was determined by chemiluminescence immunoassay (CLIA). An automatic analyzer Architect 4100 ci ABBOTT (Abbott Diagnostics Inc., Park City, IL, USA) was used to perform the analyses.

The homeostatic model assessment of insulin resistance index (HOMA-IR) was calculated using the formula developed by Matthews et al., 1985 [7].

HOMA-IR = (Insulin[μIU/mL] × Glucose [mmol/L])/22.5

### 2.5. Statistics

Data were analyzed using Statistica 13 (Dell Inc., Round Rock, TX, USA). Distribution normality was assessed via the Shapiro–Wilk test. Inter-group differences were evaluated using one-way analysis of variance (ANOVA) for independent groups, followed by Tukey’s post hoc test to identify significant differences between workers at different mining levels. Spearman’s rank correlation analysis and the Mann–Whitney U test were employed for bivariate associations. Effect size (ES) for the one-way ANOVA comparing the three groups was expressed as Cohen’s f, with values of 0.10, 0.25, and 0.40 interpreted as small, medium, and large effects, respectively [25]. Independent predictors of serum 25(OH)D concentration were identified using stepwise multiple linear regression. The statistical significance of individual predictors and changes in the coefficient of determination (R^2^; ΔR^2^) were assessed at successive steps. Data are presented as mean (SD), and statistical significance was set at *p* < 0.05.

## 3. Results

Among the study participants, 9 reported vitamin D supplementation at the lowest over-the-counter dose available in pharmacies (400 IU/10 µg), used without a physician’s recommendation. Moreover, 10 participants reported current cigarette smoking, including 3 individuals smoking up to 10 cigarettes daily, 6 smoking approximately one pack per day, and 1 smoking more than one pack per day. Individual exposure to sunlight was not assessed. Blood samples were obtained from all miners within the same week and at a similar time of day in order to minimize the potential effect of diurnal variability on the analyzed parameters. Finally, the results of 62 miners (only males) were subjected to a statistical analysis. The detailed anthropometric characteristics of the respondents, duration of mine employment, and cardiorespiratory fitness are shown in Table 1.

The examined group of miners was characterized by increased body weight, regardless of the mining level at which they worked. Seventeen participants (27%) were overweight, and 25 people (40%) were obese based on BMI. Due to the fact that the examined people worked physically, the increased BMI could be conditioned by a large muscle mass. Body composition analysis, however, showed a significant proportion of fat mass (FM) > 22%, with normal muscle mass content. Only miners working at level 2 had a normal fat mass content. Cardiorespiratory fitness as measured by VO_2max_ indicates an average level. The lowest value was recorded for miners working on the surface. Statistical analysis showed statistical significance in the difference in VO_2max_ values between miners working at different mining levels (*p* < 0.05). Miners working at level 1 were also characterized by the highest body weight, BMI, fat content, and VFA (Visceral Fat Area) index.

In each of the study groups, an elevated mean glucose concentration (>100 mg/dL) was found in as many as 29 subjects (47%). The calculated HOMA-IR index in 14 people (22%) was >2.5 and was the highest, similar to the glucose level in the group of miners working on the surface. Total cholesterol was high regardless of the study group. However, the concentration of vitamin D was considered insufficient (<30 ng/mL) for the entire group of miners, regardless of the mining level at which they work (Table 2).

Correlation analysis in the overall study group showed that VO_2max_ was inversely associated with body weight (Spearman’s R = −0.539, *p* < 0.05). Body weight, in turn, was positively associated with fasting glucose concentration (R = 0.290, *p* < 0.05), insulin concentration (R = 0.404, *p* < 0.05), and HOMA-IR (R = 0.417, *p* < 0.05). Furthermore, HOMA-IR was inversely associated with VO_2max_ (R = −0.503, *p* < 0.05) and positively associated with visceral fat area (VFA; R = 0.488, *p* < 0.05) and fat mass (FM; R = 0.491, *p* < 0.05). In the univariate analysis, serum 25(OH)D concentration was significantly associated with vitamin D supplementation (Mann–Whitney U test: U = 41.0; Z = −3.31; *p* = 0.0009), positively correlated with HDL cholesterol (R = 0.382; *p* = 0.0067), and inversely correlated with HOMA-IR (R = −0.357; *p* = 0.0119).

Stepwise multiple linear regression was performed to identify independent correlates of serum 25(OH)D concentration. Vitamin D supplementation, HDL cholesterol, and HOMA-IR were entered as candidate predictors (Table 3).

In the first step, vitamin D supplementation was retained in the model and showed a significant positive association with serum 25(OH)D concentration (β = 0.505, B = 19.64, SE = 4.89, t = 4.01, *p* < 0.001). This initial model was statistically significant (R = 0.505, R^2^ = 0.255, adjusted R^2^ = 0.239, F(1,47) = 16.101, *p* < 0.001), indicating that supplementation alone accounted for 25.5% of the variance in serum vitamin D levels. In the second step, HDL cholesterol was added to the model, resulting in a significant improvement in model fit. The two-predictor model remained statistically significant (R = 0.597, R^2^ = 0.356, adjusted R^2^ = 0.328, F(2,46) = 12.734, *p* < 0.001), with HDL contributing an additional 10.1% of explained variance (ΔR^2^ = 0.101). In this model, both vitamin D supplementation (β = 0.456, B = 17.73, SE = 4.65, t = 3.81, *p* < 0.001) and HDL cholesterol (β = 0.322, B = 0.334, SE = 0.124, t = 2.69, *p* = 0.010) were independently associated with serum 25(OH)D concentration. In the third step, HOMA-IR was evaluated as an additional predictor. Its inclusion increased the coefficient of determination from R^2^ = 0.356 to R^2^ = 0.396, corresponding to an additional 4.0% of explained variance (ΔR^2^ = 0.039). However, this improvement did not reach statistical significance (*p* = 0.094). The regression coefficient for HOMA-IR was negative (β = −0.212, B = −2.39), suggesting an inverse association with serum 25(OH)D concentration after adjustment for supplementation and HDL cholesterol, although this relationship was not statistically significant.

## 4. Discussion

The aim of this study was to investigate the association between serum 25(OH)D concentration and markers of glucose and lipid metabolism in salt mine workers. Overall, the findings provide partial support for the initial hypothesis that lower 25(OH)D levels are associated with insulin resistance and metabolic disturbances in miners. In the stepwise multiple regression analysis, vitamin D supplementation emerged as the strongest predictor of serum 25(OH)D concentration, explaining 25.5% of its variance. After inclusion of HDL cholesterol, the explained variance increased to 35.6% (ΔR^2^ = 10.1%). The addition of HOMA-IR further increased the coefficient of determination to 39.6%, corresponding to an additional 3.9% of explained variance; however, this increment did not reach statistical significance. Taken together, these findings suggest that, in this cohort, vitamin D status was more closely related to supplementation and HDL cholesterol than to HOMA-IR.

Although HOMA-IR was not retained as an independent predictor in the final model, its inverse association with serum 25(OH)D in the univariate analyses remains noteworthy. This pattern suggests that lower vitamin D levels tended to co-occur with greater insulin resistance, but that this relationship may have been attenuated after adjustment for supplementation and lipid-related factors. Such an interpretation is biologically plausible, as vitamin D has been implicated in pancreatic β-cell function, calcium homeostasis, insulin receptor expression, and peripheral insulin action [26]. Clinical studies have also suggested that vitamin D supplementation may improve insulin sensitivity and reduce HOMA-IR, particularly in individuals with vitamin D deficiency [27]. However, more recent meta-analyses indicate that, although lower 25(OH)D concentrations are generally associated with higher HOMA-IR in observational studies, the results of intervention trials remain inconsistent, and the magnitude of the effect appears to depend on baseline vitamin D status, metabolic phenotype, supplement dose, and treatment duration [28,29].

By contrast, HDL cholesterol remained an independent predictor of serum 25(OH)D concentration in the final model, suggesting that higher vitamin D status in the studied miners co-occurred with a more favorable lipid profile. This observation is consistent with epidemiological studies and meta-analyses showing that higher circulating 25(OH)D levels are associated with lower odds of low HDL-C and with an overall more favorable lipid profile [30,31,32,33]. Several mechanisms may underlie this relationship, including the effects of vitamin D on inflammation, HDL/ApoA1 function, reverse cholesterol transport, and insulin sensitivity [33]. In this respect, our results appear to support a clearer link between vitamin D status and lipid metabolism than between vitamin D status and insulin resistance assessed by HOMA-IR.

The findings regarding thyroid function were less conclusive. No statistically significant association was observed between serum 25(OH)D and TSH, suggesting that thyroid axis activity was not a major determinant of vitamin D status in this cohort. Nevertheless, this issue warrants attention, as in the descriptive analysis, workers from the lowest mining level showed both the highest TSH values and the highest 25(OH)D concentrations. The available literature on the relationship between vitamin D and thyroid function remains heterogeneous. Some studies have linked vitamin D deficiency with higher TSH levels, thyroid autoimmunity, or altered thyroid hormone profiles [30,31,32], whereas more recent critical reviews suggest that these associations are inconsistent and often attenuate after adjustment for confounding factors [34,35]. Therefore, the relationship between 25(OH)D and TSH in this group should be interpreted with caution.

Body composition also deserves consideration. In the present study, serum 25(OH)D was not significantly associated with BMI, fat mass (FM), muscle mass (MM), or visceral fat area (VFA) in the univariate analyses, although the direction of these associations was generally inverse. Surface workers, however, were characterized by the highest BMI, FM, and VFA, indicating a less favorable metabolic profile in this subgroup. This observation is relevant because excess body weight, particularly central adiposity, has repeatedly been associated with lower 25(OH)D concentrations, possibly due to sequestration of vitamin D in adipose tissue, volumetric dilution, and chronic low-grade inflammation [36,37,38,39]. Recent evidence also suggests that visceral adiposity may be more closely related to vitamin D status than BMI alone, and that low vitamin D levels may coexist with less favorable muscle-related parameters [39]. In our study, these associations did not reach statistical significance, which may reflect the relatively small sample size, the all-male study population, and the potentially confounding effect of vitamin D supplementation.

The relationship between serum 25(OH)D and physical fitness was likewise inconclusive. Vitamin D concentration showed only a borderline positive tendency in relation to VO_2max_, whereas earlier analyses demonstrated an inverse association between VO_2max_ and body weight. This is in line with the observation that surface workers not only had a less favorable body composition profile but also lower cardiorespiratory fitness. Existing literature suggests that the association between vitamin D and cardiorespiratory fitness is biologically plausible and generally positive, but relatively modest in strength, while intervention studies have not consistently shown an improvement in VO_2max_ following vitamin D supplementation [29,40,41,42,43,44,45]. It may therefore be assumed that, in this occupational group, cardiorespiratory fitness reflects overall metabolic health more closely than vitamin D status per se.

Age and employment duration were not clearly associated with serum 25(OH)D concentration in our study. Within a relatively homogeneous occupational cohort, current behavioral and environmental factors, such as supplementation, sun exposure outside work, and overall metabolic status, may be more important determinants of vitamin D status than age or job seniority alone. Nevertheless, long-term work in an environment with limited exposure to ultraviolet B radiation may still contribute to a persistently suboptimal vitamin D status over time.

These findings should also be interpreted in the broader context of miners’ occupational health. Earlier stages of our analysis showed that surface workers had a less favorable body composition profile and that the mean cardiorespiratory fitness level in the entire group was unsatisfactory. Considered together with elevated BMI, high total cholesterol, and the observed tendency toward an inverse association between 25(OH)D and HOMA-IR, these findings may indicate an increased cardiometabolic risk burden in this population. This is particularly relevant in mine workers, in whom working conditions, occupational stress, lifestyle factors, and reduced sunlight exposure may jointly aggravate metabolic risk. Studies conducted in miners and in other groups working underground or in enclosed environments suggest that these workers are at increased risk of vitamin D deficiency [46,47,48]. For this reason, extending routine occupational prevention to include a more comprehensive assessment of cardiometabolic risk, including serum 25(OH)D, insulin resistance indices, and body composition parameters, appears justified.

Overall, our findings indicate that the relationship between vitamin D status and metabolic disturbances in miners is complex. In this cohort, it was most clearly reflected by supplementation status and HDL cholesterol, whereas the association with HOMA-IR, although present in simple analyses and directionally consistent with the hypothesis, did not remain independent after adjustment. Given the cross-sectional design, these findings should be interpreted cautiously and should not be regarded as evidence of causality.

## 5. Conclusions

In this cohort of salt mine workers, insufficient vitamin D status coexisted with excess body weight, dyslipidemia, and impaired glucose homeostasis. Serum 25(OH)D concentration was primarily associated with vitamin D supplementation and HDL cholesterol, whereas its inverse relationship with HOMA-IR did not remain statistically significant after adjustment for the other variables. These findings suggest that the assessment of vitamin D status together with selected metabolic markers may be relevant in the occupational health evaluation of this professional group.

## 6. Limitations

This study has several limitations that should be considered when interpreting the findings. First, its cross-sectional design precludes causal inferences regarding the relationship between vitamin D status and metabolic alterations. Second, the relatively small sample size (n = 62) limits statistical power and reduces the generalizability of the findings. Third, the study included only male participants, which limits the applicability of the results to female populations. However, it should be noted that the number of women employed in the mining industry, particularly in underground work, is low, and no women were present in the study population. In addition, several potentially important confounding factors were not fully controlled for, including dietary intake, dose and duration of vitamin D supplementation, physical activity outside work, individual sunlight exposure, and seasonal variation, all of which may influence serum 25(OH)D concentrations as well as metabolic parameters. Another limitation is the absence of a non-exposed control group, which makes it difficult to determine whether the observed alterations are specific to miners or reflect broader population trends. Moreover, the occupational setting itself should be taken into account, as underground work performed under artificial lighting and with limited exposure to natural sunlight may substantially affect vitamin D status. Future studies should therefore include larger and more diverse populations, including women, in order to improve external validity. Longitudinal and interventional studies are needed to better clarify the relationship between vitamin D deficiency and metabolic outcomes. In particular, randomized controlled trials assessing the effects of vitamin D supplementation in occupational groups working underground or in environments with limited sunlight exposure would be especially valuable. Further research should also account for key lifestyle-related and environmental confounders and explore the biological mechanisms linking vitamin D with insulin signaling, inflammation, and adipose tissue function. Finally, incorporating vitamin D assessment into occupational health screening programs may help identify workers at increased cardiometabolic risk and support the development of preventive strategies.

## Figures and Tables

**Table 1:** Participants’ anthropometric characteristics, duration of mine employment, cardiorespiratory fitness, (mean (SD)).

	Total (*n* = 62)	Group 1 (*n* = 13)	Group 2 (*n* = 16)	Group 3 (*n* = 33)	ANOVA *p*-Value
Age (yr)	41.98 (7.68)	43.54 (8.64)	42.56 (7.08)	41.09 (7.69)	0.7305 (ES: 0.103)
Work seniority (yr)	15.10 (8.78)	12.92 (7.75)	15.84 (9.99)	15.61 (8.66)	0.7732 (ES: 0.094)
Body weight (kg)	87.24 (14.47)	93.39 (13.03)	82.12 (15.22)	87.31 (14.09)	0.0584 (ES: 0.318)
Body height (cm)	175.51 (6.12)	178.71 (6.77)	175.03 (4.75)	174.49 (4.98)	0.0991 (ES: 0.286)
BMI (kg/m^2^)	28.32 (4.43)	29.29 (4.18)	26.86 (5.08)	28.65 (4.15)	0.2408 (ES: 0.222)
VO_2max_ (mL/kg/min)	34.82 (8.49)	27.77 (6.17)	36.43 (9.76)	36.82 (7.28)	0.0047 (ES: 0.446)
FM (kg)	22.63 (8.88)	25.23 (8.34)	20.73 (9.56)	22.47 (8.80)	0.9186 (ES: 0.054)
MM (kg)	61.70 (6.49)	64.78 (5.38)	59.27 (6.22)	61.59 (6.67)	0.0561 (ES: 0.320)
VFA	10.61 (4.38)	11.61 (3.48)	10.00 (5.00)	10.48 (4.46)	0.4634 (ES: 0.163)

Group 1—surface workers; Group 2—underground workers employed at depths greater than 750 m below ground level; Group 3—underground workers employed at depths of 750 m or less below ground level. Data are presented as mean (SD), BMI—body mass index, VO_2max_—maximal oxygen uptake, FM- fat mass, MM- muscle mass, VFA—visceral fat tissue.

**Table 2:** Biochemical indices for the entire group of men involved in the study (mean (SD)).

	Total (*n* = 62)	Group 1 (*n* = 13)	Group 2 (*n* = 16)	Group 3 (*n* = 33)	ANOVA *p*-Value
Glucose (mg/dL)	104.04 (16.38)	109.25 (29.33)	102.55 (7.90)	102.93 (13.49)	0.9172 (ES: 0.054)
Insulin (µIU/mL)	10.51 (13.67)	9.20 (3.85)	9.00 (4.52)	11.88 (18.79)	0.7658 (ES: 0.095)
HOMA-IR	2.25 (1.28)	2.43 (1.03)	2.28 (1.18)	2.16 (1.47)	0.5237 (ES: 0.149)
TC (mg/dL)	217.52 (52.38)	210.00 (24.41)	214.80 (43.43)	221.81 (62.33)	0.8618 (ES: 0.071)
HDL-C (mg/dL)	52.90 (13.97)	49.60 (11.62)	54.27 (13.92)	53.37 (15.05)	0.5862 (ES: 0.135)
LDL-C (mg/dL)	136.63 (43.65)	133.00 (28.19)	132.00 (35.20)	140.56 (52.56)	0.9203 (ES: 0.053)
TG (mg/dL)	141.12 (88.72)	136.30 (61.97)	142.40 (62.37)	142.19 (109.57)	0.7130 (ES: 0.107)
25(OH)D (ng/mL)	26.36 (14.21)	23.36 (7.05)	24.80 (12.25)	28.54 (17.26)	0.9186 (ES: 0.054)
TSH (mU/L)	2.66 (8.01)	1.33 (0.67)	1.50 (0.89)	3.81 (11.06)	0.9729 (ES: 0.031)

Group 1—surface workers; Group 2—underground workers employed at depths greater than 750 m below ground level; Group 3—underground workers employed at depths of 750 m or less below ground level. Data are presented as mean (SD), HOMA-IR—homeostatic model assessment of insulin resistance index, TC—total cholesterol, HDL-C—high density lipoprotein cholesterol, LDL-C—low density lipoprotein cholesterol. TG—triglycerides, 25(OH)D—25-hydroxyvitamin D, TSH—thyroid-stimulating hormone.

**Table 3:** Stepwise linear regression models assessing the impact of selected biochemical, anthropometric, and physical fitness characteristics of salt mine workers on serum 25(OH)D concentration.

Variable	R^2^	β	Adjusted R^2^	F	*p* Value
Step 1	0.255		0.239	16.101	≤0.001 **
Vit. D suppl. Yes-1, No-0		0.505			≤0.001 **
Step 2	0.356		0.328	12.734	≤0.001 **
Vit. D suppl. Yes-1, No-0		0.456			≤0.001 **
HDL-C (mg/dL)		0.322			≤0.05 *
Step 3	0.396		0.355	9.818	≤0.001 **
Vit. D suppl. Yes-1, No-0		0.413			≤0.001 **
HDL-C (mg/dL)		0.265			≤0.05 *
HOMA-IR		−0.212			0.094

* *p* < 0.05; regression coefficients are significant at the 0.05 level (two–tailed). ** *p* < 0.001; regression coefficients are significant at the 0.01 level (two–tailed). R^2^, coefficient of determination; β, standardized linear regression coefficient; F, test statistic for overall model significance. HOMA-IR—homeostatic model assessment of insulin resistance index, HDL-C—high-density lipoprotein cholesterol.

## Data Availability

The datasets generated and analyzed during the current study are not publicly available due to institutional restrictions, but are available from the corresponding author on reasonable request.

## Abbreviations

The following abbreviations are used in this manuscript:

25(OH)D	25-hydroxyvitamin D
BIA	Bioelectric Impedance Analysis
BMI	Body Mass Index
CLIA	Chemiluminescence Immunoassay
CMIA	Carbonyl Metalloimmunoassay
CRP	C-reactive Protein
ES	Effect Size
FM	Fat Mass
HDL-C	High-Density Lipoprotein Cholesterol
HOMA-IR	Homeostatic Model Assessment for Insulin Resistance
IFG	Impaired Fasting Glucose
LDL-C	Low-Density Lipoprotein Cholesterol
MetS	Metabolic Syndrome
MM	Muscle Mass
PTH	Parathyroid Hormone
TC	Total Cholesterol
TG	Triglycerides
TSH	Thyroid-Stimulating Hormone (Thyrotropin)
VDR	Vitamin D Receptor
VFA	Visceral Fat Area (Visceral Fat Tissue)
VO_2max_	Maximal Oxygen Consumption
